# Capacidad de detección de sobrecrecimiento bacteriano o metanogénico intestinal de los test de aliento para intolerancia a lactosa y fructosa en población adulta

**DOI:** 10.1515/almed-2024-0040

**Published:** 2024-05-15

**Authors:** Emilio José Laserna Mendieta, Verónica Martín Dominguez, Irene Pérez Lucendo, Inmaculada Granero Cremades, Raquel Ferreirós Martínez, Tomás Álvarez Malé, María Ángeles Sanz De Benito, Cecilio Santander

**Affiliations:** Unidad de Investigación, Servicio de Digestivo, 203291 Hospital General de Tomelloso, Tomelloso, Ciudad Real, España; Servicio de Análisis Clínicos, 16517 Hospital Universitario de La Princesa, Madrid, España; Instituto de Investigación Sanitaria de La Princesa, Madrid, España; Instituto de Investigación Sanitaria de Castilla-La Mancha, Toledo, España; Centro de Investigación Biomédica en Red Enfermedades Hepáticas y Digestivas (CIBERehd), Madrid, España; Servicio de Aparato Digestivo, 16517 Hospital Universitario de La Princesa, Madrid, España; Departamento de Medicina, Universidad Autónoma de Madrid (UAM), Madrid, España

**Keywords:** malabsorción/intolerancia a fructosa, malabsorción/intolerancia a lactosa, sobrecrecimiento bacteriano intestinal, sobrecrecimiento metanogénico intestinal, test de aliento espirado

## Abstract

**Objetivos:**

Los test de aliento espirado (TAE) son la principal herramienta diagnóstica en la evaluación de la malabsorción/intolerancia a fructosa (IF) y lactosa (IL) y para la detección del sobrecrecimiento bacteriano o metanógenico intestinal (SCBI/SCMI). En ocasiones, los TAE-IF/IL muestran hallazgos sugerentes de la presencia de SCBI o SCMI, pero los estudios que evalúan la fiabilidad de éstos son escasos. Nuestro objetivo es analizar la sensibilidad y especificidad de los TAE-IF/IL en la detección de SCBI y la concordancia en la identificación de SCMI.

**Métodos:**

Estudio observacional retrospectivo donde se seleccionaron entre 652 TAE realizados mediante cromatografía de gases aquellos TAE-IF/IL y TAE-SCBI hechos en un mismo paciente en un plazo máximo de 6 semanas.

**Resultados:**

Se encontraron 146 TAE de 67 pacientes adultos que cumplieron el criterio de selección. La especificidad para la detección de SCBI fue mejor para el TAE-IL que para TAE-IF (93,8 vs. 72,7 %). La sensibilidad fue más alta para el TAE-IF (60,0 vs. 28,6 %), porque se observó un mayor porcentaje de IF que de IL entre los pacientes con SCBI positivo (70 vs. 29 %). Para SCMI, la concordancia fue del 100 % para TAE-IL y hubo un 27 % de falsos negativos en TAE-IF.

**Conclusiones:**

Los hallazgos de SCBI o SCMI encontrados en el TAE-IL son altamente concordantes con los obtenidos en la prueba TAE-SCBI, mientras que los TAE-IF presentan en torno a un 27 % de falsos positivos en la detección de SCBI y otro 27 % de falsos negativos en la identificación de SCMI.

## Introducción

El sobrecrecimiento bacteriano intestinal (SCBI) es un problema digestivo funcional producido por un crecimiento excesivo de la microbiota bacteriana en el intestino delgado y se acompaña de síntomas como hinchazón, flatulencia, dolor abdominal y diarrea o estreñimiento [1, 2]. Estos síntomas pueden ser debidos a una alteración en la permeabilidad y motilidad intestinal, un mayor grado de inflamación o de activación inmune, una malabsorción de nutrientes o la fermentación de sustratos en el intestino delgado [3].

En ocasiones, este sobrecrecimiento es debido a un exceso de microbiota productora de metano (CH_4_) o metanogénica (SCMI) [4]. Estos organismos metanogénicos, principalmente *Methanobrevibacter smithii* [5], no son bacterias sino arqueas, de ahí que se haya propuesto un término diferente para referirse a su sobrecrecimiento [1]. El SCMI se ha asociado con estreñimiento, debido al efecto inhibitorio que ejerce el CH_4_ en el peristaltismo intestinal [6]. Un amplio estudio retrospectivo mostró que el SCMI es una condición distinta del SCBI, y que el exceso de CH_4_ es más frecuente en pacientes ancianos y afecta menos a la malabsorción de nutrientes como la vitamina B12 [7]. Además, el tratamiento antibiótico indicado también es diferente, pues la mejoría en los síntomas de los pacientes con SCMI fue más alta al emplear una combinación de neomicina y rifaximina que con rifaximina en monoterapia como en el SCBI [8, 9].

Aunque la prueba diagnóstica considerada como “patrón oro” para el SCBI es el aspirado yeyunal con posterior cultivo, la dificultad en su realización al ser una técnica invasiva, su coste y la falta de consenso sobre el número de unidades formadoras de colonias por mL que se debe usar como punto de corte, hacen que su uso clínico sea anecdótico [3]. Así, la prueba más utilizada para la evaluación de un posible SCBI o SCMI es, por su bajo coste, su carácter no invasivo, facilidad de realización y amplia disponibilidad en muchos laboratorios, el test de aliento espirado (TAE) en el que se miden niveles de hidrógeno (H_2_), metano (CH_4_) y dióxido de carbono (CO_2_), éste último empleado como factor corrector y para comprobar la correcta realización de la toma de muestras.

Existen diferentes guías y consensos para la realización e interpretación de los TAE, incluyendo ciertas condiciones previas en la preparación de los pacientes que van a realizar el test, destacando las guías de expertos americana [10] y europea [11]. Estas guías varían en los puntos de corte que deben emplearse en la interpretación de los TAE-SCBI, ya que la guía americana propone la existencia de un SCBI cuando ocurre un aumento de H_2_ ≥20 partes por millón (ppm) respecto al valor basal antes del minuto 90, y la guía europea solo menciona un aumento temprano del H_2_ ≥10–12 ppm respecto al valor basal sin especificar un intervalo temporal. La guía americana además propone que una concentración de CH_4_ elevada (≥10 ppm) es indicativa de SCMI, mientras que la europea no menciona nada al respecto. Los sustratos más recomendados son 50–75 g de glucosa o 10–20 g de lactulosa, aunque en España, otro sustrato semejante a la lactulosa, el lactitol, se emplea frecuentemente en la evaluación del SCBI por ser ambos azúcares no absorbibles. Un reciente meta-análisis mostró un ligero mejor rendimiento diagnóstico para la glucosa sobre la lactulosa [12].

Como cualquier prueba, los TAE también presentan limitaciones. Entre ellas, que se trata de una medida indirecta, que su interpretación puede verse afectada por un tránsito orocecal acelerado/disminuido (de forma fisiológica o por la presencia de cirugías, fármacos y/o patologías que lo puedan afectar) o la falta de correlación con los resultados de los cultivos de aspirado de intestino delgado [13]. Además, dado que las arqueas metanogénicas emplean el H_2_ como sustrato para la producción de CH_4_, los niveles de H_2_ suelen ser bajos cuando existe SCMI y, por tanto, es imprescindible que los instrumentos empleados para medir los gases espirados en los TAE proporcionen la concentración de CH_4_ [14].

Por otro lado, la malabsorción/intolerancia a carbohidratos, como la fructosa (IF) o lactosa (IL), puede estar relacionada con la presencia de SCBI y producir una sintomatología similar, ya que el SCBI puede conllevar la disminución de su absorción debido a la afectación de las vellosidades y/o destrucción de las enzimas de la mucosa intestinal [15, 16]. Así, no es raro en la práctica clínica solicitar un TAE para IF y/o IL en los mismos pacientes en los que se investiga un SCBI/SCMI. Estos TAE-IF/IL pueden revelar información sobre un posible SCBI o SCMI, cuando la elevación de H_2_ se produce de forma precoz o cuando se observan concentraciones elevadas de CH_4_, respectivamente [17].

Aunque la guía de consenso americana recomienda realizar en primer lugar un TAE-SCBI antes de los TAE para malabsorción/intolerancia a carbohidratos [10], la realidad es que, por motivos prácticos y debido a la inespecificidad de los síntomas, a veces se solicitan varios TAE en la misma consulta. Por eso, saber qué coincidencias existen entre los TAE-IF/IL con el TAE-SCBI podría ser útil para evitar repeticiones de TAE, nuevas consultas y retrasos diagnósticos, y con ello disminuir costes. Sin embargo, muy pocos estudios han abordado esta cuestión, y siempre con un enfoque centrado en evaluar los casos positivos para IF/IL que podrían ser causados por SCBI [18, 19].

El objetivo del presente estudio es evaluar si los hallazgos sugestivos de SCBI y SCMI encontrados en los TAE-IF/IL realizados en población adulta son coincidentes con el resultado obtenido en los TAE-SCBI, estableciendo así la sensibilidad y especificidad de los primeros en la detección de SCBI y la concordancia en la identificación de SCMI.

## Materiales y métodos

### Pacientes

Estudio observacional retrospectivo donde se recogieron los resultados de los TAE realizados por práctica clínica habitual en el Hospital Universitario de La Princesa (HULP) entre febrero de 2020 y abril de 2023 (n=652), y se seleccionaron aquellos que se realizaron en un mismo paciente en un plazo máximo de 6 semanas (42 días), sin mediar cambios de tratamiento entre los TAE. El tiempo mínimo que transcurrió entre diferentes TAE fue de 1 semana, al especificarse como tal en las instrucciones dadas a los pacientes en los que se solicitó más de un TAE. Todos los TAE fueron realizados en población adulta mayor de 16 años.

De estos pacientes, se extrajeron los siguientes datos: sexo, edad en el momento de realizar el primer TAE, tipo de TAE realizado, diferencia en días entre el primer y el último TAE realizado y resultado del TAE. En aquellos donde la interpretación facultativa fue que existió una mala preparación (por niveles basales de H_2_ y/o CH_4_ elevados junto con disminución de los mismos en tiempos posteriores) [20] o una incorrecta toma de muestras (niveles de CO_2_ inferiores al 2 %), no se incluyeron en el análisis final.

Los datos de los pacientes fueron tratados de forma anónima y obtenidos exclusivamente de aquellos registrados en el Sistema Informático de Laboratorio, sin acceder a las historias clínicas, cumpliendo la legislación vigente y siguiendo los acuerdos de la Declaración de Helsinki.

### Realización de los TAE

Los TAE fueron suministrados por la empresa Isomed Pharma SL (Madrid, España) y consistieron en la toma de una muestra basal de aire espirado y a continuación la ingesta de un sustrato: 25 g de fructosa (para IF), 25 g de lactosa (para IL) y 10 g de lactitol (para SCBI). Después, se recogió una muestra de aire espirado cada 25 minutos hasta llegar a los 175 minutos (8 muestras en total). Se incluyeron tanto TAE realizados por el paciente en su propio domicilio (siguiendo unas instrucciones escritas proporcionadas por el personal médico) como TAE realizados en el hospital bajo la supervisión de personal de enfermería.

En los tubos de aire espirado se midieron los niveles de H_2_, CH_4_ y CO_2_ en un cromatógrafo de gases BreathTracker SC Analyzer (QuinTron, Milwaukee, WI, EEUU) disponible en el HULP. El rango de medición fue 2–150 ppm para el H_2_, 2–75 ppm para el CH_4_ y 0,1–7,0 % para el CO_2_. Los valores de CO_2_ se emplearon como factor corrector de la concentración de H_2_ y CH_4_ para un valor de 5,5 %, cálculo que el equipo realiza de forma automática. Un gas de concentración conocida suministrado por Isomed Pharma SL se empleó para las calibraciones y controles de calidad del cromatógrafo.

### Interpretación de los TAE y análisis de la concordancia

La interpretación de resultados de los TAE se realizó en base a las recomendaciones de las guías clínicas con ligeras modificaciones [10, 11]. Así, se consideró que un TAE-IF/IL es sugerente de presencia de SCBI cuando existió un incremento ≥20 ppm de H_2_ respecto al valor basal en el minuto 90 o antes (en nuestro caso, en el minuto 75 que corresponde a la cuarta toma de muestra) y que es sugestivo de SCMI cuando los valores de CH_4_ fueron ≥10 ppm (en nuestro caso, cuando dicha condición se da en todos los tiempos). Los mismos criterios se aplicaron para definir la positividad para SCBI y SCMI en el TAE-SCBI. Cuando se detectó un SCMI, los TAE-SCBI no se incluyeron en el análisis de la concordancia para la detección de SCBI, dada la influencia que tiene la producción de CH_4_ en la concentración de H_2_ al ser éste el sustrato empleado en la generación del CH_4_, resultando así en la mayoría de casos en curvas planas de H_2_ [4]. De forma paralela, los TAE-IF/IL se consideraron positivos para IF/IL cuando se produjo un incremento ≥20 ppm de H_2_ o de ≥10 ppm de CH_4_ respecto al valor basal en al menos un punto de la curva, siguiéndose en este caso el criterio recomendado por ambas guías.

Cuando se obtuvo un resultado positivo tanto en el TAE-SCBI como en el TAE-IF/IL, la malabsorción/intolerancia a carbohidratos podría ser secundaria a la presencia de SCBI y se calculó el porcentaje de estos casos respecto al total de positivos para SCBI. La distinción entre malabsorción o intolerancia se realizó en función de la escala validada de Casellas *et al.*, que incluye 5 síntomas (diarrea, dolor abdominal, vómitos, borborigmos y gases/flatulencias) valorados del 0 al 10 según su intensidad, aceptándose que existe intolerancia cuando la suma de los mismos es igual o mayor a 7 puntos o malabsorción cuando es menor a 7 puntos [21].

Para la concordancia entre los TAE-IF/IL y el TAE-SCBI en la detección de SCBI, se construyeron tablas de contingencia en las que se calculó la sensibilidad, especificidad, valor predictivo positivo (VPP) y valor predictivo negativo (VPN) con sus correspondientes intervalos de confianza (IC) al 95 % usando el programa GraphPad Prism versión 5.0 (GraphPad Software, San Diego, CA, EEUU). Para la concordancia entre TAE-IL/IF y el TAE-SCBI en la detección de SCMI, se calculó el porcentaje de coincidencia.

## Resultados

### Datos demográficos de los pacientes y características de los TAE

De los 652 TAE realizados en nuestro hospital durante el periodo del estudio, 146 cumplieron el criterio de selección, correspondientes a 67 TAE-SCBI, 47 TAE-IF y 32 TAE-IL. Estos TAE se realizaron a 67 pacientes, 82 % mujeres y con una edad media 47,8±16,9 años (rango 17,7–85,6).

**Figura 1:**
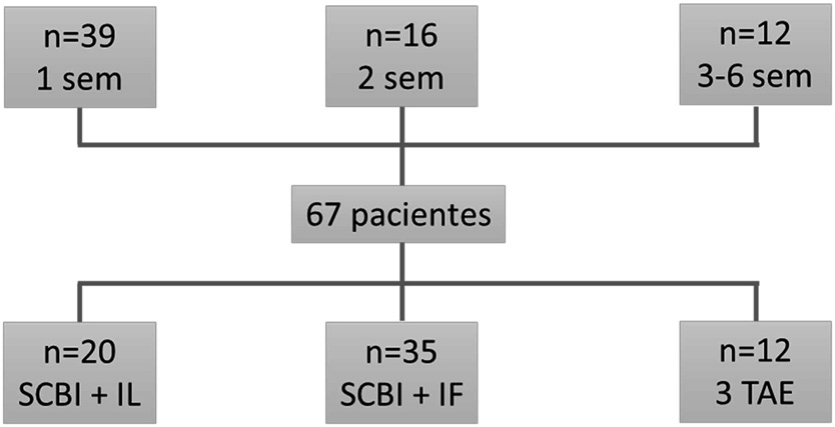
Características de los test de aliento espirado (TAE) incluidos en el análisis final. Se especifica el tipo de TAE: Intolerancia a fructosa (IF), intolerancia a lactosa (IL) o sobrecrecimiento bacteriano intestinal (SCBI), así como el tiempo transcurrido en semanas entre el primer y último TAE realizado en un mismo paciente.

Respecto al tipo de TAE y las diferencias de tiempo entre ellos, los resultados se muestran en la Figura 1. La mayoría de pacientes (52 %) realizaron un TAE-IF y TAE-SCBI, mientras que en solo un 18 % se llevaron a cabo los tres tipos de TAE. El intervalo de tiempo más frecuente entre TAE fue de una semana (58 %), seguido de 2 semanas (24 %) y finalmente de entre 3 y 6 semanas (18 %).

De los pacientes con un resultado positivo para el TAE-IF un 52,9 % presentaron además intolerancia, mientras que este porcentaje fue mayor entre los positivos para el TAE-IL al ser un 77,8 %.

### Concordancia entre los TAE-IF/IL y TAE-SCBI para la detección de SCBI

Para evaluar la capacidad de detección de SCBI de los TAE-IF/IL se compararon los resultados de 32 TAE-IF y 23 TAE-IL.

Para el TAE-IF, se obtuvo una sensibilidad del 60,0 % (IC: 26,2–87,8 %) y una especificidad del 72,7 % (IC: 49,8–89,3 %), con un VPP del 50,0 % (IC: 21,1–78,9 %) y un VPN del 80,0 % (IC: 56,3–94,3 %) (Tabla 1). Así, en esta comparación se observó que en un 27,3 % de los TAE-IF sugestivos de SCBI, dicho hallazgo no se observó en el TAE-SCBI. En los 10 pacientes con SCBI, 7 presentaron además un resultado positivo para IF.

**Tabla 1:** Tabla de contingencia con los resultados obtenidos para los pacientes en los que se realizaron el test de aliento espirado de intolerancia a fructosa (TAE-IF) y el de sobrecrecimiento bacteriano (TAE-SCBI).

	TAE-SCBI positivo	TAE-SCBI negativo	Total
TAE-IF sugestivo de SCBI	6	6	12
TAE-IF no sugestivo de SCBI	4	16	20
Total	10	22	32

Para el TAE-IL, el número de falsos positivos fue menor (6,2 %), lo que se tradujo en una mayor especificidad. Los resultados de esta comparación se muestran en la Tabla 2, siendo la sensibilidad del 28,6 % (IC: 3,7–71,0 %), la especificidad del 93,8 % (IC: 69,8–99,8 %), con un VPP del 66,7 % (IC: 9,4–99,2 %) y un VPN del 75,0 % (IC: 50,9–91,3 %). De los 7 pacientes con SCBI, en solo 2 se observó además un resultado positivo para IL.

**Tabla 2:** Tabla de contingencia con los resultados obtenidos para los pacientes en los que se realizaron el test de aliento espirado de intolerancia a lactosa (TAE-IL) y el de sobrecrecimiento bacteriano (TAE-SCBI).

	TAE-SCBI positivo	TAE-SCBI negativo	Total
TAE-IL sugestivo de SCBI	2	1	3
TAE-IL no sugestivo de SCBI	5	15	20
Total	7	16	23

Estos valores de sensibilidad, especificidad, VPP y VPN para la detección de SCBI en los TAE-IF/IL aparecen recogidos de forma resumida en la Tabla 3.

**Tabla 3:** Resumen de los valores de sensibilidad, especificidad, valor predictivo positivo y valor predictivo negativo para la detección de sobrecrecimiento bacteriano intestinal en los test de aliento espirado (TAE) de intolerancia a fructosa y lactosa. Los valores entre paréntesis representan los intervalos de confianza al 95 %.

	TAE fructosa	TAE lactosa
Sensibilidad	60,0 % (26,2–87,8 %)	28,6 % (3,7–71,0 %)
Especificidad	72,7 % (49,8–89,3 %)	93,8 % (69,8–99,8 %)
Valor predictivo positivo	50,0 % (21,1–78,9 %)	66,7 % (9,4–99,2 %)
Valor predictivo negativo	80,0 % (56,3–94,3 %)	75,0 % (50,9–91,3 %)

Entre los pacientes incluidos en estos análisis, hay 8 en los que se realizaron las 3 pruebas. En 6 de ellos la concordancia entre las pruebas fue completa, mientras que en 2 pacientes se detectó elevación de H_2_ en el TAE-IF en el minuto 50 sugiriendo la presencia de un SCBI, pero el TAE-SCBI fue negativo, lo cual si fue concordante con lo observado en el TAE-IL.

### Concordancia entre los TAE-IF/IL y TAE-SCBI en la evaluación de SCMI

En 20 pacientes se obtuvo en el TAE-SCBI un resultado indicativo de presencia de SCMI. El número de TAE-IF/IL para comparar fue de 15 y 9, respectivamente, porque en 4 de estos pacientes se realizaron tanto TAE-IF como TAE-IL. No se observó ningún TAE-IF/IL que mostrara SCMI y donde no se detectara esa condición en el TAE-SCBI (Tabla 4).

**Tabla 4:** Concordancia entre los resultados obtenidos para el test de aliento espirado de intolerancia a fructosa (TAE-IF) y lactosa (TAE-IL) en los pacientes con un test de aliento espirado para sobrecrecimiento bacteriano (TAE-SCBI) para la evaluación del sobrecrecimiento metanogénico intestinal (SCMI).

	TAE-SCBI positivo para SCMI	TAE-SCBI negativo para SCMI	Concordancia
TAE-IF sugestivo de SCMI	11	0	43/47 (91,5 %)
TAE-IF no sugestivo de SCMI	4	32	
TAE-IL sugestivo de SCMI	9	0	32/32 (100 %)
TAE-IL no sugestivo de SCMI	0	23	

El porcentaje de concordancia para la evaluación de SCMI fue del 100 % para el TAE-IL y del 91,5 % para TAE-IF, debido a que éste último no fue capaz de detectar el SCMI en 4 casos, lo que se traduce en un 27 % de falsos negativos. En los 4 pacientes en los que se realizaron ambos test, se detectó el SCMI tanto en el TAE-IF como en el TAE-IL.

## Discusión

Los TAE representan una herramienta útil para evaluar trastornos funcionales en pacientes con síntomas digestivos inespecíficos como hinchazón, dolor abdominal o flatulencias, por su bajo coste y facilidad de realización [11]. Así, un estudio con 1.230 pacientes en los que se descartó patología digestiva estructural mediante endoscopia y exploraciones radiológicas, demostró que un 45 % de ellos presentaban SCBI, IF y/o IL detectados mediante TAE [22].

El presente estudio arroja resultados muy relevantes sobre los hallazgos encontrados en los TAE-IF/IL relacionados con SCBI, aportando más datos a la escasa bibliografía disponible al respecto, y proporciona, por primera vez, resultados sobre la concordancia en la detección de SCMI. Así, hemos observado que el TAE-IF presenta un 27 % de falsos positivos para detección de SCBI, es decir, elevaciones a tiempos cortos de H_2_ que no se correspondieron con un SCBI al realizar el TAE-SCBI con lactitol, mientras que dicho porcentaje fue solo de un 6 % para el TAE-IL, lo que resultó en una alta especificidad. Para la identificación de SCMI, el TAE-IL también presentó unos resultados óptimos al mostrar una concordancia del 100 %, mientras que hubo un 27 % de falsos negativos para el TAE-IF.

La identificación de SCMI es posible gracias al desarrollo de cromatógrafos de gases capaces de medir CH_4_ además de H_2_. El porcentaje de pacientes con SCMI podría tener una prevalencia del 30–60 % cuando se determina mediante TAE [23], siendo en nuestro centro el 57 % de todos los sobrecrecimientos detectados con este método [24]. En nuestra experiencia, al usar lactitol como sustrato, los SCMI se manifiestan como una meseta donde el CH_4_ es elevado desde el momento basal, presentando valores ≥10 ppm en todos los puntos de la curva del TAE. La guía americana, en cambio, basada en el empleo de lactulosa o glucosa, proponen la identificación de un SCMI cuando el CH_4_ ≥10 ppm en cualquier punto de la curva [10]. Por el contrario, la guía europea no hace ninguna recomendación para la detección del SCMI [11]. Con lactitol, el CH_4_ incrementa sus niveles paralelamente al H_2_, por lo que es frecuente encontrar CH_4_ ≥10 ppm en el minuto 100 o posteriores. Nuestra interpretación sí es coincidente con un reciente estudio que observó una alta sensibilidad (86 %) y especificidad (100 %) para detectar SCMI en pacientes con muestras basales con CH_4_ ≥10 ppm en los TAE-SCBI de glucosa y lactulosa [25].

Nuestro estudio no solo es el primero en evaluar la concordancia de los TAE-IF/IL con el TAE-SCBI en la detección de SCMI, sino que, según nuestro conocimiento, ningún estudio ha evaluado de forma tan concisa y precisa el rendimiento de los TAE-IF/IL para la detección de SCBI. Un primer estudio ya identificó al SCBI como una causa que incrementaba la probabilidad de presentar IL en pacientes con diarrea crónica, pero sin ahondar en la comparación entre ambos [26]. Estudios previos solo han observado que el porcentaje de positivos para IL o intolerancia al sorbitol, pero no para IF, es mayor cuando los pacientes dan positivo en el TAE-SCBI [18] o que el SCBI es una condición frecuente (75 %) que causa IL detectada únicamente por TAE y no por test de tolerancia oral [19]. Un estudio asiático encontró que la IL secundaria a SCBI era de un 15 % [27], un porcentaje algo inferior al observado en nuestro estudio (29 %). Por último, un estudio reciente ha observado un mayor porcentaje de casos de SCBI (28 % frente a un 7 % en controles) en pacientes con deficiencia de lactasa comprobada mediante biopsia [28]. Así, nuestros resultados aportan datos novedosos en lo referente a la IF posiblemente secundaria a SCBI, que fue del 70 %, o la menor discordancia con el TAE-SCBI en los hallazgos de SCBI observados en el TAE-IL (6 %) en comparación con el TAE-IF (27 %).

Las principales fortalezas de nuestro estudio es que emplea los TAE recogidos a lo largo de 3 años, realizados en un mismo cromatógrafo de gases y en pacientes en los que transcurrió un breve periodo de tiempo para la realización de los diferentes TAE. Como limitaciones, podemos mencionar que la elevación de H_2_ podría deberse en algunos pacientes a un tránsito intestinal acelerado, aunque en esos casos se afectaría por igual el resultado del TAE-SCBI y de los TAE-IF/IL. Además, el empleo de lactitol y la recogida de aire espirado cada 25 min que impide medir los gases en el minuto 90, son características del TAE-SCBI realizado en nuestro hospital que no se ajustan completamente a las recomendaciones de las guías americana o europea [10, 11]. Como ocurre en la mayoría de centros, no se realizaron cultivos de aspirado duodenal por práctica clínica habitual, por lo que no se pudo comparar los resultados del TAE-SCBI con los de esta prueba de referencia; por este motivo, los resultados expuestos son aplicables a la comparación entre los TAE-IF/IL con el TAE-SCBI empleando como sustrato el lactitol y serían necesarios más estudios con mayor número de pacientes y con otros sustratos (glucosa o lactulosa) para confirmar nuestros hallazgos. Tampoco se ha medido la producción de sulfuro de hidrógeno, que podría ser el responsable de la presencia de curvas planas sin elevación de H_2_ ni CH_4_ en 5 pacientes y que se interpretaron como negativas para SCBI y SCMI.

Como conclusión, nuestro estudio demuestra que la detección en el TAE-IL de un SCBI o SCMI tiene una alta concordancia con el resultado obtenido en el TAE-SCBI, por lo que no sería necesario la realización del mismo, con el consiguiente retraso en el diagnóstico e instauración de tratamiento. En cambio, el TAE-IF presenta menor concordancia con el TAE-SCBI, ya que en un 27 % de los casos el SCBI detectado será un falso positivo y el SCMI no se identificará en otro 27 % de los TAE-IF. Estos resultados refuerzan la importancia de la formación de los profesionales en la interpretación óptima de los TAE que, junto con su adecuada indicación, permiten incrementar las tasas de diagnósticos correctos, tratamientos eficaces y mejorar la relación coste-eficacia de estas pruebas.
